# The Redox–Inflammation Axis in Atherosclerosis and Ischemic Stroke: Mechanisms, Biomarkers, and Translational Challenges

**DOI:** 10.3390/ijms27156705

**Published:** 2026-07-27

**Authors:** Cezary Gaczyński, Aleksandra Polikowska, Małgorzata Goszka, Natalia Serwin, Urszula Łacek, Adrianna Jerzyk, Rafał Heryć, Barbara Dołęgowska, Elżbieta Cecerska-Heryć

**Affiliations:** 1 Department of Laboratory Medicine, Pomeranian Medical University, 70-111 Szczecin, Poland; 72787@student.pum.edu.pl (C.G.); aleksandra.polikowska@gmail.com (A.P.); malgosia@goszka.pl (M.G.); natalia.serwin@pum.edu.pl (N.S.); 77312@student.pum.edu.pl (U.Ł.); 72464@student.pum.edu.pl (A.J.); barbara.dolegowska@pum.edu.pl (B.D.); 2 Clinic of Cardiac Surgery, Pomeranian Medical University, 70-111 Szczecin, Poland; rafher87@wp.pl

**Keywords:** atherosclerosis, acute ischemic stroke, oxidative stress, inflammation, redox signaling, reactive oxygen species, Nrf2, NLRP3 inflammasome, biomarkers, precision cardiovascular medicine

## Abstract

Cardiovascular diseases (CVDs) remain the leading cause of mortality worldwide and are driven by the interplay between oxidative stress and chronic inflammation. Although these processes have been extensively investigated, their integration into a unified mechanistic framework and their translational relevance for biomarker-guided precision medicine remain insufficiently addressed. This narrative review integrates mechanistic, biomarker, and translational perspectives on the redox–inflammation axis in atherosclerosis and acute ischemic stroke, critically evaluating oxidative and inflammatory biomarkers while highlighting current challenges and emerging therapeutic opportunities. Reactive oxygen and nitrogen species (ROS/RNS) promote endothelial dysfunction, lipid oxidation, and activation of redox-sensitive pathways, including NF-κB, Nrf2/Keap1, JAK/STAT, and the NLRP3 inflammasome, resulting in vascular inflammation and injury. Biomarkers such as hs-CRP, IL-6, MPO, MDA, F2-isoprostanes, oxLDL, and antioxidant enzyme activity have demonstrated associations with cardiovascular risk, disease severity, and clinical outcomes, and therefore show diagnostic and prognostic potential. However, with the exception of selected inflammatory biomarkers, most oxidative stress biomarkers have not yet been sufficiently validated for routine clinical use because of biological variability, limited specificity, and insufficient analytical standardization. Disappointing outcomes of antioxidant supplementation further reflect the complexity of redox biology and the dual physiological and pathological roles of ROS. Here, we highlight that future progress in cardiovascular medicine will require a shift from non-specific antioxidant supplementation to precision redox modulation, integrating multimarker profiling, system biology, multi-omics, and artificial intelligence and machine-learning approaches to improve cardiovascular risk stratification and personalized therapy.

## 1. Introduction

Cardiovascular diseases (CVDs) remain the leading cause of mortality worldwide, accounting for approximately one-third of all global deaths [1]. Despite major advances in prevention, diagnosis, and treatment, the global burden of CVD continues to rise and is projected to increase by nearly 90% between 2025 and 2050, reaching an estimated 35.6 million deaths annually [2]. Although traditional risk factors, including hypertension, dyslipidemia, diabetes, obesity, smoking, and physical inactivity, are well established, growing evidence indicates that oxidative stress and chronic inflammation represent fundamental molecular mechanisms driving endothelial dysfunction, vascular remodeling, atherosclerosis, and ischemic injury.

Over the past two decades, considerable progress has been made in identifying biomarkers, advanced imaging techniques, and novel therapeutic strategies for CVDs. Nevertheless, early diagnosis, accurate risk stratification, and effective prevention of disease progression remain major clinical challenges. Biomarkers, imaging modalities, and artificial intelligence- and machine learning-based approaches are increasingly recognized as complementary tools supporting precision cardiovascular medicine [3,4,5].

Although oxidative stress and inflammation have been extensively investigated in cardiovascular diseases, these processes are frequently discussed separately or within disease-specific contexts. Existing narrative reviews typically focus on individual signaling pathways, biomarkers, or therapeutic interventions, whereas comprehensive frameworks integrating mechanistic, biomarker, and translational perspectives remain limited. Furthermore, the inconsistent results of antioxidant-based clinical trials highlight the complexity of redox biology and underscore the need for a more integrated understanding of the redox–inflammation axis.

Unlike previous reviews, which generally address oxidative stress, inflammation, biomarkers, or antioxidant therapies as separate aspects of cardiovascular pathology, the present review proposes a unified conceptual framework in which oxidative stress and inflammation are viewed as a dynamic, self-amplifying redox–inflammation axis. Within this framework, mechanistic signaling pathways, biomarker performance, and translational challenges are integrated to explain both disease progression and the limited clinical success of conventional antioxidant therapies. Because atherosclerosis and acute ischemic stroke represent two of the most extensively investigated cardiovascular disorders in which oxidative stress and inflammation play central pathogenic roles, this review primarily focuses on these disease entities. This targeted approach enables a comprehensive discussion of the molecular basis of the redox–inflammation axis, biomarker utility, and emerging therapeutic strategies while maintaining sufficient analytical depth. By integrating mechanistic insights with biomarker assessment and translational evidence, this review provides a comprehensive translational framework that bridges the gap between experimental redox biology and clinical cardiovascular medicine.

### 1.1. Conceptual Framework: The Redox–Inflammation Axis in Cardiovascular Diseases

Oxidative stress and inflammation are no longer regarded as independent pathological processes but rather as two interconnected components of a dynamic biological network that underlies the initiation and progression of cardiovascular diseases (CVDs) [6,7,8,9]. Under physiological conditions, reactive oxygen and nitrogen species (ROS/RNS) function as essential signaling molecules that regulate endothelial homeostasis, vascular tone, mitochondrial function, immune responses, and cellular adaptation [10,11,12,13,14]. However, when ROS production exceeds the capacity of endogenous antioxidant systems or when redox signaling becomes dysregulated, oxidative stress develops, leading to endothelial dysfunction, lipid oxidation, extracellular matrix remodeling, and vascular injury [6,7,8,15].

Multiple enzymatic and non-enzymatic sources contribute to excessive ROS generation in cardiovascular diseases. These include mitochondrial dysfunction, NADPH oxidase (NOX), xanthine oxidase (XOR), uncoupled endothelial nitric oxide synthase (eNOS), and myeloperoxidase (MPO), all of which have been implicated in vascular oxidative damage [5,16,17,18,19,20]. Recent reviews further emphasize that oxidative stress affects cardiovascular disease progression through interconnected molecular networks involving inflammation, apoptosis, fibrosis, metabolic imbalance, and endothelial dysfunction [21,22]. Increased ROS production activates redox-sensitive signaling pathways, including NF-κB, MAPK, JAK/STAT, and the NLRP3 inflammasome, thereby enhancing transcription of pro-inflammatory mediators, such as IL-1β, IL-6, TNF-α, and IFN-γ [11,12,13,14,16,23,24,25].

Inflammation, in turn, further amplifies oxidative stress by activating macrophages, neutrophils, lymphocytes, platelets, and endothelial cells, which generate additional ROS and reactive nitrogen species via NOX enzymes, MPO, and mitochondrial pathways [6,7,8,9,11]. This creates a self-perpetuating feed-forward loop in which oxidative stress promotes inflammation, while inflammation further enhances oxidative damage. The relative contribution of these mechanisms differs among cardiovascular diseases and depends on the predominant involvement of endothelial cells, vascular smooth muscle cells, immune cells, cardiomyocytes, or microglia, highlighting the context-dependent nature of the redox–inflammation axis [22].

Importantly, this evolving concept extends beyond the traditional view of oxidative stress as merely excessive accumulation of ROS. Current evidence indicates that oxidative stress should be considered a disturbance of tightly regulated redox signaling rather than a simple imbalance between oxidants and antioxidants [12,24]. The Nrf2/Keap1 pathway is particularly important in this context because it coordinates endogenous antioxidant responses and intersects with inflammatory signaling; however, its effects in cardiovascular diseases may be context-dependent rather than uniformly protective [26]. This paradigm helps explain why conventional antioxidant supplementation has generally failed to demonstrate consistent cardiovascular benefit in large clinical trials, despite strong experimental evidence supporting the pathogenic role of oxidative stress [9,27,28].

**Figure 1:**
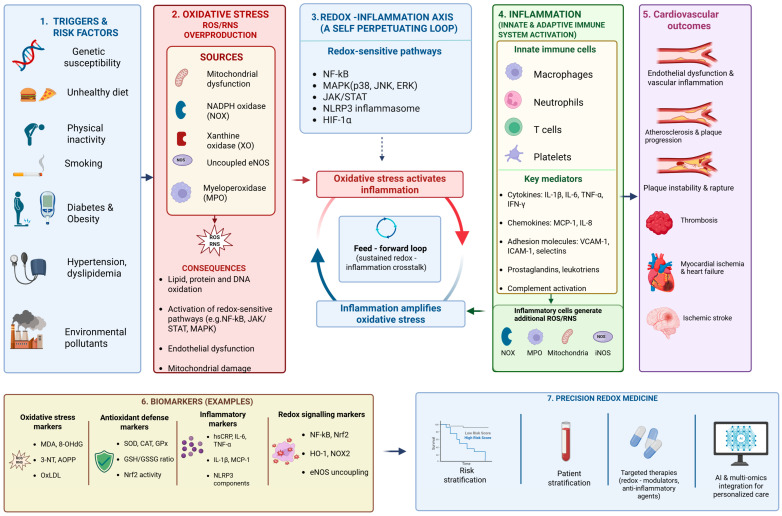
Integrated overview of the redox–inflammation axis in atherosclerosis and acute ischemic stroke. Classical cardiovascular risk factors promote excessive production of reactive oxygen and nitrogen species (ROS/RNS) through multiple enzymatic sources, including mitochondrial dysfunction, NADPH oxidases (NOX), xanthine oxidase (XO), uncoupled endothelial nitric oxide synthase (eNOS), and myeloperoxidase (MPO). Excessive ROS/RNS activate redox-sensitive signaling pathways, including NF-κB, MAPK, JAK/STAT, the NLRP3 inflammasome, and HIF-1α, thereby initiating a self-amplifying redox–inflammation feedback loop. Activation of innate and adaptive immune cells further exacerbates oxidative stress by releasing cytokines, chemokines, adhesion molecules, prostaglandins, leukotrienes, and complement components. Sustained crosstalk between oxidative stress and inflammation promotes endothelial dysfunction, atherosclerotic plaque progression, plaque instability, thrombosis, myocardial ischemia, heart failure, and acute ischemic stroke. Representative oxidative stress, antioxidant, inflammatory, and redox signaling biomarkers may facilitate cardiovascular risk stratification, while targeted redox-modulating therapies, biomarker-guided patient stratification, and integration of artificial intelligence (AI) and multi-omics approaches represent promising directions for precision cardiovascular medicine. Created by the authors using BioRender. Cecerska-Heryć, E. (2026) https://BioRender.com/pdosl6l (accessed on 15 May 2026).

This integrated redox–inflammation framework provides the conceptual basis for understanding the molecular mechanisms discussed throughout this review. It also supports the development of multimarker strategies and precision medicine approaches that combine oxidative stress biomarkers, inflammatory mediators, imaging techniques, multi-omics, and artificial intelligence for improved cardiovascular risk stratification and individualized therapeutic interventions [21,29] (Figure 1).

### 1.2. Lifestyle-Related Risk Factors in Cardiovascular Diseases

Lifestyle-related factors, including unhealthy diet, physical inactivity, obesity, diabetes, smoking, and excessive alcohol consumption, are major contributors to the development and progression of cardiovascular disease (CVD) [18,30,31]. Beyond their well-established metabolic and hemodynamic effects, these factors promote oxidative stress, chronic low-grade inflammation, and endothelial dysfunction, thereby accelerating vascular injury and atherosclerosis [6,7,8,9].

A healthy dietary pattern rich in vegetables, fruits, whole grains, and fish, together with regular physical activity, has consistently been linked to lower cardiovascular risk, whereas smoking, obesity, diabetes, and excessive alcohol consumption increase cardiovascular morbidity and mortality [10,18,20,27,28,31,32,33,34,35,36,37,38,39,40,41,42,43,44]. These risk factors share molecular mechanisms, including increased ROS generation, impaired antioxidant defenses, inflammatory activation, and endothelial dysfunction, ultimately promoting lipid oxidation, plaque development, and cardiovascular events [6,7,8,9].

**Figure 2:**
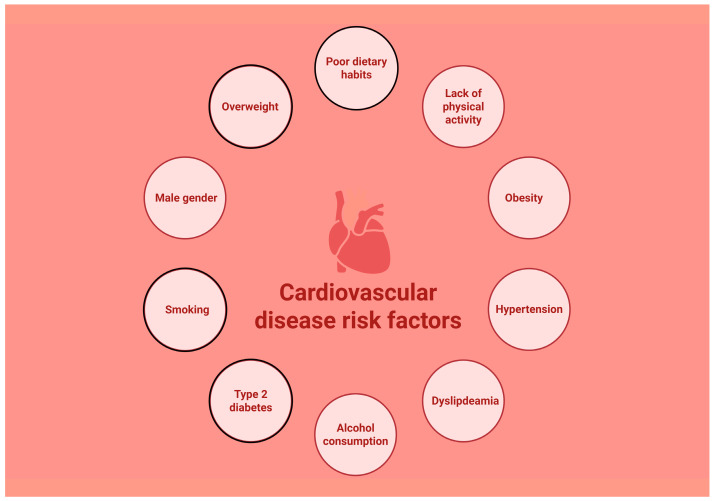
Cardiovascular risk factors. Created in BioRender. Cecerska-Heryć, E. (2026) https://BioRender.com/70lkq7m (accessed on 15 May 2026) [30,45].

Because these processes converge on the redox–inflammation axis, lifestyle factors should be viewed not only as independent cardiovascular risk factors but also as major modulators of redox homeostasis and vascular inflammation. Figure 2 provides a schematic overview of the principal lifestyle-related cardiovascular risk factors.

## 2. Aim of This Study

This paper aims to review, discuss, and critically analyze the current scientific literature on the role of inflammation and oxidative stress in the pathogenesis of cardiovascular diseases, with particular emphasis on the importance of biomarkers of these processes in diagnostics, monitoring, and prevention. The starting point for the analysis was the hypothesis that inflammation and oxidative stress play key roles in the development and progression of cardiovascular diseases, and that associated biomarkers may be valuable tools to support diagnostic and therapeutic activities. Therefore, a research question was posed: Can biomarkers of inflammation and oxidative stress be used effectively for the diagnosis, monitoring, and prevention of cardiovascular diseases?

## 3. Literature Search Strategy

This article was prepared as a narrative review to synthesize current evidence on the role of the redox–inflammation axis in cardiovascular diseases, with particular emphasis on mechanistic pathways, biomarkers, and translational challenges. As a narrative review, the literature search was designed to identify the most relevant mechanistic, translational, and clinical evidence rather than to provide an exhaustive systematic synthesis of evidence or a formal risk-of-bias assessment.

A literature search was conducted between 20 November 2024 and 20 May 2025 using the PubMed, Google Scholar, and ScienceDirect databases. The search strategy combined Medical Subject Headings (MeSH) and free-text keywords related to disease mechanisms, biomarkers, and therapeutic strategies, including “cardiovascular diseases”, “atherosclerosis”, “ acute ischemic stroke”, “oxidative stress”, “inflammation”, “redox signaling”, “reactive oxygen species”, “reactive nitrogen species”, “molecular mechanisms”, “mechanistic pathways”, “endothelial dysfunction”, “Nrf2”, “NF-κB”, “JAK/STAT”, “NLRP3 inflammasome”, “biomarkers”, “antioxidants”, “redox modulation”, and “precision medicine”. Rather than aiming for exhaustive retrieval, the search strategy was designed to identify the most relevant and methodologically robust publications that directly addressed the objectives of this narrative review. Keywords were combined using Boolean operators (AND/OR) to identify studies that address the interactions among oxidative stress, inflammation, biomarkers, and cardiovascular diseases.

Studies published in English were considered. Preference was given to original research articles, meta-analyses, systematic reviews, and high-quality narrative reviews published within the last 10 years, although earlier landmark studies were included when considered essential for understanding fundamental mechanisms.

Publications were selected based on their scientific relevance, methodological quality, and contribution to understanding the molecular mechanisms linking oxidative stress, inflammation, biomarkers, and therapeutic strategies in cardiovascular diseases, with particular emphasis on endothelial dysfunction, redox-sensitive signaling pathways, immune activation, and translational relevance.

Inclusion criteria

Studies were included if they:

- Were published in English;
- Were original research articles, systematic reviews, meta-analyses, or high-quality narrative reviews;
- Investigated molecular and cellular mechanisms underlying the redox–inflammation axis, including redox-sensitive signaling pathways (e.g., Nrf2, NF-κB, JAK/STAT, and the NLRP3 inflammasome);
- Evaluated diagnostic or prognostic biomarkers or translational aspects of cardiovascular diseases, with particular emphasis on atherosclerosis and acute ischemic stroke;
- Provided mechanistic, experimental, preclinical, or clinical evidence relevant to the objectives of this review.

Exclusion criteria

Studies were excluded if they:

- Were conference abstracts, editorials, letters to the editor, comments, or case reports;
- Were duplicate records;
- Were not directly related to cardiovascular diseases, the redox–inflammation axis, or the molecular mechanisms underlying cardiovascular pathology;
- Focused exclusively on non-cardiovascular conditions without translational relevance to cardiovascular pathology;
- Lacked sufficient scientific quality or did not contribute substantially to the objectives of this review.

**Figure 3:**
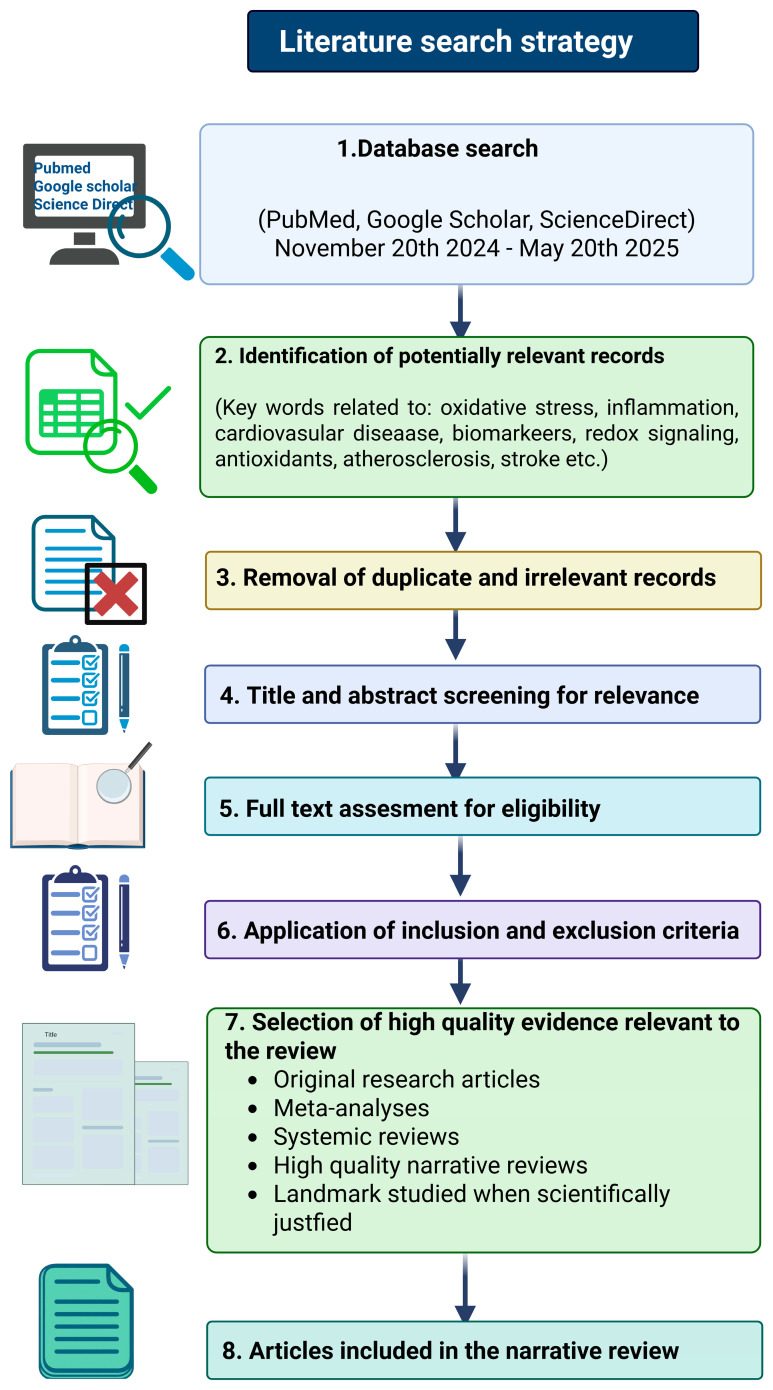
PRISMA—inspired flow diagram illustrating the literature identification and selection process for this narrative review. Publications were identified through searches of PubMed, Google Scholar, and ScienceDirect, screened for relevance, assessed for eligibility, and selected according to predefined inclusion and exclusion criteria. Created in BioRender. Cecerska-Heryć, E. (2026) https://BioRender.com/1u68gfg (accessed on 15 May 2026).

When several publications addressed the same topic, priority was given to recent high-quality studies, landmark publications, meta-analyses, and systematic reviews, while older studies were included when they represented seminal findings or were essential to understanding fundamental molecular mechanisms underlying cardiovascular disease pathogenesis. The literature search strategy is shown in Figure 3.

## 4. Oxidative Stress and Free Radicals

Oxidative stress is defined as a phenomenon resulting from an imbalance between the production and accumulation of free radicals—reactive oxygen and nitrogen species- in cells and tissues, and the body’s ability to detoxify these reactive forms [24]. Free radicals, on the other hand, are atoms or molecules containing one or more unpaired electrons. The odd number of electrons makes them unstable and highly reactive [11,46]. Generated in moderate or low quantities, ROS and RNS have many beneficial effects and participate in the body’s physiological processes; however, in excessive amounts, they generate oxidative stress, leading to cell damage and, consequently, various pathological conditions [11].

Enzymatic and non-enzymatic reactions form these substances, and their sources can be classified as exogenous or endogenous. Endogenous sources of free radicals include immune cell activation, inflammation, infections, cancer, excessive physical activity, and aging. Conversely, among exogenous sources of free radicals, exposure to environmental pollutants (e.g., heavy metals), certain drugs (e.g., cyclosporine, gentamicin), cigarette smoke, and alcohol is highlighted [12,24].

### 4.1. Major Sources of Reactive Oxygen Species in Cardiovascular Diseases

Reactive oxygen species (ROS) in cardiovascular tissues originate from multiple enzymatic and non-enzymatic sources, each contributing differently to physiological redox signaling and cardiovascular pathology. The major enzymatic sources include mitochondria, NADPH oxidases (NOX), xanthine oxidoreductase (XOR), and uncoupled endothelial nitric oxide synthase (eNOS), whose relative contributions vary with disease stage, cell type, and inflammatory microenvironment.

Mitochondria are a major intracellular source of ROS through electron leakage from the electron transport chain, primarily generating superoxide anion (O_2_•^−^) [14]. Mitochondrial ROS (mtROS) play a central role in ischemia–reperfusion injury, endothelial dysfunction, and activation of redox-sensitive signaling pathways.

Another major source is the NADPH oxidase (NOX) family, particularly NOX1, NOX2, and NOX4, which generate superoxide in response to inflammatory and mechanical stimuli [11,23]. NOX-derived ROS contribute to endothelial activation, vascular remodeling, leukocyte recruitment, and amplification of inflammatory signaling.

Xanthine oxidoreductase (XOR) also contributes substantially to oxidative stress in cardiovascular diseases by producing both superoxide and hydrogen peroxide, particularly under hypoxic and inflammatory conditions [11]. In parallel, endothelial nitric oxide synthase (eNOS) may become uncoupled under oxidative conditions, shifting from nitric oxide (NO) production toward superoxide generation. This process simultaneously increases oxidative stress and reduces NO bioavailability, thereby aggravating endothelial dysfunction [13,47,48].

Reactive oxygen and nitrogen species comprise a heterogeneous group of molecules that differ markedly in their sources, biological functions, and pathological consequences [6,7,9,23,49]. Superoxide anion (O_2_•^−^), generated primarily by NOX enzymes, mitochondria, and xanthine oxidase, represents the initial ROS from which several secondary reactive species are formed [6,9,23,49]. Hydrogen peroxide (H_2_O_2_), owing to its relative stability and membrane permeability, serves as an important intracellular redox signaling molecule regulating endothelial function, vascular tone, angiogenesis, and inflammatory responses [6,7,23]. In contrast, hydroxyl radicals (•OH) are extremely reactive and induce irreversible oxidative damage to lipids, proteins, and DNA [6,7,9]. Peroxynitrite (ONOO^−^), generated by the reaction of superoxide with nitric oxide, promotes protein nitration, endothelial dysfunction, mitochondrial injury, and blood–brain barrier disruption, particularly during ischemia–reperfusion injury [9,49,50,51,52,53]. The contribution of individual ROS-generating systems also differs among cardiovascular cell types. In endothelial cells, NOX enzymes and uncoupled eNOS are major sources of oxidative stress, promoting endothelial dysfunction and leukocyte adhesion [7,9,47,54,55,56]. In vascular smooth muscle cells, NOX-derived ROS stimulate proliferation, migration, and vascular remodeling [54,56,57,58]. Macrophages generate ROS primarily through NOX2 and mitochondria, thereby amplifying inflammatory signaling, foam cell formation, and plaque instability [7,49,54,57,59]. Neutrophils contribute to oxidative injury through the respiratory burst and myeloperoxidase activity, whereas platelet-derived ROS promote thromboinflammation and platelet activation [50,54,56,57,58]. In acute ischemic stroke, activated microglia become an important source of ROS and inflammatory mediators, contributing to blood–brain barrier disruption and secondary neuronal injury [50,51,52,53,60].

### 4.2. Redox-Sensitive Signaling Pathways Linking Oxidative Stress and Inflammation

The interaction between oxidative stress and inflammation is mediated by redox-sensitive signaling pathways that regulate gene expression and immune activation.

Among these, the NF-κB pathway plays a central role. ROS activate NF-κB signaling, leading to the transcription of pro-inflammatory cytokines such as IL-1β, IL-6, and TNF-α, thereby amplifying inflammatory responses [17,49,61,62,63,64,65,66].

In contrast, the Nrf2/Keap1 pathway represents the principal endogenous defense mechanism against oxidative stress. Under physiological conditions, Nrf2 is retained in the cytoplasm through its interaction with Keap1 and is continuously degraded by the proteasome. Increased oxidative or electrophilic stress disrupts this interaction, allowing Nrf2 to translocate into the nucleus, where it binds antioxidant response elements (AREs) and induces the transcription of numerous cytoprotective genes, including superoxide dismutase (SOD), catalase (CAT), glutathione peroxidase (GPx), heme oxygenase-1 (HO-1), and NAD(P)H quinone oxidoreductase-1 (NQO1Beyond regulating antioxidant defenses, Nrf2 modulates mitochondrial function, endothelial homeostasis, inflammatory signaling, and regulated cell death pathways, including ferroptosis and pyroptosis, by maintaining intracellular redox homeostasis, limiting lipid peroxidation, and suppressing inflammasome activation. Consequently, dysregulation of Nrf2 signaling has been implicated in endothelial dysfunction, atherosclerosis, ischemia–reperfusion injury, and other cardiovascular disorders. Recent studies further indicate that Nrf2 plays an important regulatory role in ferroptosis and pyroptosis by maintaining intracellular redox homeostasis, limiting lipid peroxidation, and suppressing inflammasome activation [49,58,67,68,69].

Taken together, accumulating evidence identifies the Nrf2/Keap1 pathway as one of the most promising therapeutic targets currently under investigation in cardiovascular medicine.

The NLRP3 inflammasome represents another critical link between oxidative stress and inflammation. ROS-mediated activation of inflammatory pathways promotes the release of IL-1β and IL-18, which play key roles in vascular inflammation and atherosclerosis [16,64,70,71,72,73,74].

These pathways demonstrate that oxidative stress and inflammation are not independent processes but rather components of an integrated signaling network that drives cardiovascular pathology.

## 5. Pro-Inflammatory Cytokines

Cytokines are a diverse group of proteins regulating intercellular communication under both physiological and pathological conditions. Based on their biological functions, they are broadly classified as pro- and anti-inflammatory mediators, with pro-inflammatory cytokines playing a central role in the initiation and progression of cardiovascular inflammation [75]. Although the molecular actions of these cytokines are largely conserved across cardiovascular diseases, their relative contributions vary with disease phenotype. In atherosclerosis, IL-1β, IL-6, TNF-α, and IFN-γ promote endothelial dysfunction, leukocyte recruitment, lipid accumulation, plaque progression, and plaque destabilization [7,9,23,54,56,57,59]. In acute ischemic stroke, these cytokines contribute to blood–brain barrier disruption, neuroinflammation, cerebral edema, and secondary neuronal injury [50,51,52,53,60]. Similar inflammatory pathways have also been implicated in myocardial infarction, heart failure, and other cardiovascular disorders [23,54,56]; however, because atherosclerosis and acute ischemic stroke represent the most extensively investigated models of redox–inflammation interactions, these disease entities constitute the primary focus of the present review.

### 5.1. Interleukin-1 (IL-1) Family

The IL-1 family is a key regulator of innate and chronic inflammatory responses and includes IL-1, IL-18, and IL-36 subfamilies [74]. Major pro-inflammatory members include IL-1α, IL-1β, IL-18, IL-33, and IL-36β [70].

IL-1α and IL-1β share similar biological functions and signal through the IL-1R1 receptor. IL-1α is constitutively expressed in various cell types and is released during necrosis, initiating local inflammation [70,74]. IL-1β, produced mainly by monocytes and macrophages, is activated by caspase-1 and induces NF-κB signaling, promoting cytokine production, immune activation, and tissue remodeling processes [64,70,73,74].

IL-18, also activated by caspase-1, participates in both innate and adaptive immunity and may induce pyroptosis. It promotes Th1 responses and IFN-γ production and is associated with inflammatory and autoimmune diseases, including atherosclerosis [64,70,71,72].

IL-33 functions as an alarmin released in response to cell damage and is involved in type 2 immune responses. Its activity is tightly regulated by proteolysis and oxidation, and it signals via the ST2 receptor expressed on Th2 cells [70,76,77,78,79]. Interestingly, IL-33 may exert protective effects in atherosclerosis, whereas its soluble receptor (sST2) serves as a prognostic biomarker [70,80,81].

### 5.2. Interleukin-6 (IL-6)

IL-6 is a pleiotropic cytokine involved in inflammation, immune regulation, and metabolism. It signals via the IL-6R/gp130 receptor complex, activating the JAK/STAT pathway and inducing the synthesis of acute-phase proteins, including CRP and fibrinogen [62,64,65]. IL-6 also regulates iron metabolism via hepcidin and contributes to hematopoiesis, including platelet production, making it an important marker of systemic inflammation. Elevated circulating IL-6 concentrations have consistently been associated with increased cardiovascular risk, plaque instability, and adverse clinical outcomes following myocardial infarction and acute ischemic stroke [62,64,65].

### 5.3. Tumor Necrosis Factor Alpha (TNF-α)

TNF-α is a key pro-inflammatory cytokine produced by immune and non-immune cells. It signals through TNF-R1 and TNF-R2 receptors, activating NF-κB and MAPK pathways and promoting inflammation, endothelial activation, and tissue remodeling [17,61,63,66]. TNF-α also induces the production of other cytokines, adhesion molecules, and prothrombotic factors, contributing to sustained inflammatory responses. Persistent TNF-α activation contributes to endothelial dysfunction, ventricular remodeling, and progression of both atherosclerotic disease and heart failure [17,61,63,66].

### 5.4. Interferon Gamma (IFN-γ)

IFN-γ is a central regulator of immune responses, primarily signaling via the JAK/STAT pathway. It enhances antigen presentation, promotes Th1 differentiation, and exerts antiviral and antiproliferative effects [82,83,84]. In chronic inflammation, alternative signaling pathways may also be activated, further amplifying immune responses. In atherosclerosis, IFN-γ promotes macrophage activation and plaque instability, whereas in acute ischemic stroke it contributes to sustained neuroinflammation and secondary tissue injury [84,85].

### 5.5. Crosstalk Between Oxidative Stress and Inflammation in Cardiovascular Diseases

Oxidative stress and inflammation are tightly interconnected processes that reinforce each other, forming a self-perpetuating cycle that drives cardiovascular pathology [16,23,25,49]. During the inflammatory response, activated immune cells—particularly neutrophils and macrophages—produce large amounts of reactive oxygen and nitrogen species (ROS/RNS) as part of host defense mechanisms [16]. However, excessive or sustained production of these species leads to oxidative damage in surrounding tissues and amplifies vascular injury.

Pro-inflammatory cytokines further enhance oxidative stress. For example, interferon gamma (IFN-γ), particularly in combination with lipopolysaccharide (LPS), has been shown to increase ROS production in multiple cell types [23,84]. Similarly, interleukin-6 (IL-6) can stimulate ROS generation by inducing NADPH oxidase activity, particularly NOX4, thereby contributing to sustained oxidative imbalance [23,65].

Conversely, oxidative stress itself can initiate and propagate inflammatory signaling. Reactive species such as hydrogen peroxide (H_2_O_2_) activate transcription factors, including NF-κB, thereby increasing the expression of pro-inflammatory cytokines, chemokines, and adhesion molecules [49]. Other redox-sensitive pathways, including Nrf2 and PPAR-γ, also modulate the balance between oxidative stress and inflammation [49].

Additionally, ROS-induced lipid peroxidation products and oxidized lipoproteins act as danger-associated molecular patterns (DAMPs), further stimulating immune cell recruitment and activation [25]. This bidirectional interaction creates a feed-forward loop in which inflammation exacerbates oxidative stress, and oxidative stress amplifies inflammation, ultimately contributing to endothelial dysfunction, plaque instability, and thrombotic complications in cardiovascular diseases [8,25].

## 6. Oxidative Stress and Inflammatory Biomarkers: Limitations and Translational Challenges

Although numerous biomarkers of oxidative stress and inflammation have been proposed for cardiovascular risk assessment, their clinical applicability remains limited despite strong mechanistic rationale [86,87,88,89,90,91,92,93,94,95] The clinical significance of these biomarkers should be interpreted in light of their intended application, as biological associations with disease do not necessarily translate into diagnostic utility, prognostic value, incremental predictive performance beyond established cardiovascular risk factors, or routine clinical implementation.

Markers such as hs-CRP and IL-6 are widely used to assess systemic inflammation and have been associated with cardiovascular risk and clinical outcomes [88,91,92]. However, these markers lack specificity, as they reflect general inflammatory status rather than vascular-specific processes. For example, hs-CRP is considered a marker of inflammation accompanying atherosclerosis rather than a direct causal factor [91]. In contrast, although many oxidative stress biomarkers have shown significant associations with disease severity and prognosis, robust evidence demonstrating incremental predictive value beyond established cardiovascular risk factors remains limited for most of them.

Oxidative stress biomarkers, including malondialdehyde (MDA), F2-isoprostanes, and lipid oxidation products such as 13-HODE, provide insights into lipid peroxidation and oxidative damage [86,87,94]. Nevertheless, their clinical utility is constrained by several limitations, including variability in measurement methods, differences between biological matrices (e.g., plasma, serum, erythrocytes), and susceptibility to pre-analytical and environmental factors.

Another important limitation is the dynamic and compartment-specific nature of oxidative stress. ROS generation occurs in specific cellular compartments—such as mitochondria, vascular wall, or circulating blood cells—while most biomarkers reflect systemic changes, potentially obscuring localized pathological processes.

Furthermore, biological variability—including circadian fluctuations, metabolic status, comorbidities (e.g., diabetes, chronic kidney disease), and genetic differences in antioxidant systems—may significantly influence biomarker levels and their interpretation. These factors complicate the standardization and reproducibility of oxidative stress measurements.

Importantly, most clinical studies evaluate individual biomarkers in isolation. However, given the complexity of redox biology, a single marker is unlikely to capture the full spectrum of oxidative and inflammatory processes. This highlights the need for multimarker approaches integrating inflammatory, oxidative, and metabolic parameters to improve diagnostic accuracy and prognostic value.

Taken together, despite promising associations, current oxidative stress and inflammatory biomarkers face substantial challenges in clinical translation, underscoring the need for improved standardization, validation, and integration into precision medicine frameworks. The comparative diagnostic and prognostic utility of key oxidative stress and inflammatory biomarkers in cardiovascular diseases is shown in Table 1.

Overall, the currently available biomarkers differ substantially in their diagnostic performance, prognostic value, clinical applicability, and translational readiness. Among inflammatory biomarkers, hs-CRP is incorporated into selected cardiovascular risk assessment algorithms and clinical guidelines owing to its wide availability and standardized assays. However, its diagnostic specificity is limited because elevated concentrations occur in numerous inflammatory and infectious conditions. IL-6 provides greater mechanistic insight into vascular inflammation and may offer higher sensitivity for early inflammatory activation, yet its broader clinical implementation is hindered by biological variability and the absence of standardized diagnostic thresholds. Moreover, robust evidence demonstrating incremental predictive value beyond established cardiovascular risk factors remains limited for most oxidative stress biomarkers. In contrast, oxidative stress biomarkers, including MDA, F2-isoprostanes, oxLDL, and glutathione-related parameters, provide valuable information on redox imbalance and disease mechanisms but remain largely confined to research settings owing to methodological heterogeneity, limited assay harmonization, and insufficient prospective clinical validation. Consequently, no single biomarker currently offers sufficient sensitivity and specificity to comprehensively characterize the complex redox–inflammation axis in cardiovascular diseases. Increasing evidence therefore supports multimarker strategies integrating inflammatory, oxidative, metabolic, and imaging parameters to improve cardiovascular risk assessment and facilitate precision medicine approaches. Accordingly, most currently available biomarkers should be regarded as complementary rather than standalone tools for cardiovascular risk assessment and patient stratification.

## 7. The Role of Inflammation and Oxidative Stress in Individual Cardiovascular Diseases

### 7.1. Atherosclerosis

The following section summarizes the principal ROS sources, inflammatory pathways, representative biomarkers, current therapeutic targets, and major translational challenges associated with atherosclerosis. Atherosclerosis is a chronic inflammatory disease of large and medium-sized arteries characterized by lipid accumulation, fibrosis, and calcification within the vascular wall. It underlies major cardiovascular events, including ischemic heart disease and stroke, accounting for approximately 50% of CVD-related deaths [54,57,106].

The process is initiated by endothelial dysfunction, triggered by factors such as hypertension, hyperlipidemia, smoking, and hyperglycemia. This leads to increased vascular permeability and accumulation of low-density lipoproteins (LDL) in the intima, where they undergo oxidative modification to form oxidized LDL (oxLDL) [54,57].

OxLDL promotes the expression of adhesion molecules (VCAM-1, ICAM-1) and chemokines (e.g., MCP-1), facilitating monocyte recruitment and differentiation into macrophages. These cells internalize oxLDL via scavenger receptors, forming foam cells—hallmarks of early atherosclerotic lesions [56,57,59,99,104].

As the disease progresses, activated macrophages and T lymphocytes release pro-inflammatory cytokines, including IFN-γ, IL-1β, and TNF-α, which amplify inflammation and induce matrix metalloproteinases (MMPs), leading to extracellular matrix degradation and plaque destabilization [54,57,59]. Advanced plaques are characterized by a lipid-rich necrotic core and a thin fibrous cap, whose weakening increases the risk of rupture and subsequent thrombus formation, potentially resulting in myocardial infarction or stroke [57,107].

Calcification represents a later stage of plaque development and may contribute both to stabilization and increased vulnerability, depending on the inflammatory context [101,107]. Recent evidence highlights the importance of early intervention, particularly targeting apoB-containing lipoproteins and modulating immune responses before advanced structural changes occur [108].

IFN-γ, predominantly produced by Th1 lymphocytes, plays a key role in sustaining inflammation and promoting plaque progression by activating macrophages and increasing the expression of adhesion molecules [109].

**Figure 4:**
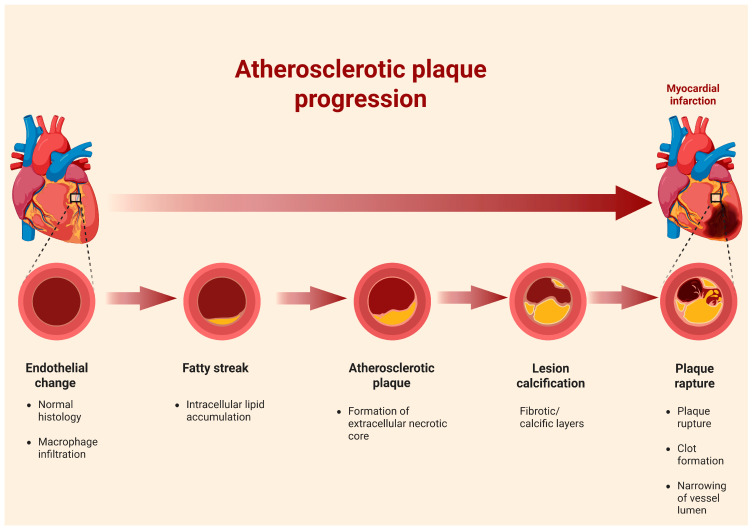
Schematic overview of the initiation and progression of atherosclerosis [57,59]. Stages of atherosclerotic plaque progression include endothelial dysfunction, fatty streak formation due to lipid accumulation, development of a lipid-rich necrotic core, lesion calcification, and plaque rupture, which can lead to thrombus formation and luminal narrowing. Created in BioRender. Cecerska-Heryć, E. (2026) https://BioRender.com/l0cg9z2 (accessed on 15 May 2026).

In summary, atherosclerosis is a multifactorial process driven by endothelial dysfunction, lipid oxidation, chronic inflammation, and vascular remodeling, underscoring the importance of early, targeted therapeutic strategies. Although numerous biomarkers and therapeutic targets have been identified, further prospective validation and biomarker standardization are required before routine clinical implementation can be recommended. The major stages of atherosclerosis development are illustrated in Figure 4, whereas the molecular mechanisms linking oxidative stress and inflammation are summarized in Figure 1.

#### Markers of Inflammation and Oxidative Stress in Atherosclerosis

Many markers of inflammation and oxidative stress are associated with the initiation and progression of atherosclerosis, providing valuable insight into the underlying pathophysiological processes. These biomarkers are widely used in both clinical and research settings to assess disease activity and cardiovascular risk.

Among inflammatory markers, high-sensitivity C-reactive protein (hs-CRP) is one of the most extensively studied. It reflects systemic inflammation and is synthesized in the liver in response to cytokines such as IL-6. Its levels increase rapidly during inflammatory processes and have been shown to predict cardiovascular events [88]. However, large meta-analyses indicate that CRP is primarily a marker of ongoing inflammation rather than a direct causal factor in atherosclerosis [91].

Interleukin-6 (IL-6), a key upstream regulator of CRP, plays a more direct role in atherosclerotic plaque progression and is more strongly associated with future cardiovascular events [92]. Additional markers, including suPAR, serum amyloid A (SAA), and myeloperoxidase (MPO), further reflect immune activation and oxidative processes. In particular, MPO contributes to LDL oxidation and plaque destabilization, thereby linking inflammation with oxidative damage [89,90,95].

Evidence from clinical studies, such as the MASCADI study, supports the coexistence of inflammatory and oxidative pathways, demonstrating elevated hs-CRP, IL-1β, and lipid oxidation products in patients with metabolic disorders and atherosclerosis [93].

Markers of oxidative stress, including malondialdehyde (MDA), F2-isoprostanes, and 13-HODE, provide insight into lipid peroxidation and redox imbalance. Increased MDA levels correlate with disease severity and myocardial injury markers such as troponin I [87], while F2-isoprostanes are considered among the most reliable indicators of oxidative damage in vivo [94]. 13-HODE, derived from linoleic acid oxidation, further links oxidative stress with lipid metabolism and foam cell formation.

In addition, oxidized low-density lipoprotein (oxLDL) represents a key biomarker linking oxidative stress with lipid metabolism and immune activation, playing a central role in foam cell formation and plaque progression [54,57]. Endothelial dysfunction is further reflected by impaired nitric oxide (NO) bioavailability, often resulting from eNOS uncoupling under oxidative conditions [13,47,48]. Moreover, intracellular redox balance can be assessed using the glutathione redox couple (GSH/GSSG ratio), which is increasingly recognized as a sensitive marker of oxidative stress [100,105,110,111].

Importantly, these biomarkers should not be interpreted in isolation but rather as interconnected components of the redox–inflammation axis, in which oxidative stress and immune activation mutually reinforce each other, driving atherosclerotic progression. This underscores the need for integrated, multimarker approaches that better capture the complexity of cardiovascular pathology and may improve risk stratification and personalized therapeutic strategies. Notably, emerging evidence suggests that compartment-specific oxidative biomarkers—particularly those derived from vascular tissue, circulating blood cells, or mitochondrial sources—may provide greater diagnostic and prognostic value than systemic markers alone, as they more accurately reflect localized redox imbalance and disease activity. Additional information on inflammatory and oxidative stress biomarkers in atherosclerosis is provided in Table 2.

### 7.2. Stroke

Stroke remains one of the leading causes of mortality and long-term disability worldwide and comprises ischemic and hemorrhagic subtypes, with acute ischemic stroke accounting for approximately 85% of all cases.

Acute ischemic stroke (AIS) is caused by vascular occlusion, leading to reduced cerebral blood flow and initiation of a complex cascade involving acidosis, blood–brain barrier disruption, oxidative stress, and inflammatory activation [51,53]. Energy failure results in dysfunction of ion pumps and excessive calcium influx, which activates proteases and endonucleases, ultimately triggering apoptosis and necrosis [50,52,53]. These events initiate a self-amplifying redox–inflammation cascade that links oxidative damage with innate immune activation and ultimately determines the extent of neuronal injury.

Neuroinflammation plays a central role in ischemic injury. Activation of glial cells and infiltration of immune cells lead to the release of pro-inflammatory cytokines, including TNF-α, IL-1β, IL-6, and IFN-γ, which amplify tissue damage [50,52,53]. IL-1β, produced early after ischemia, contributes to endothelial dysfunction and edema formation. Oxidative stress further exacerbates injury, as the brain is particularly susceptible due to high lipid content and relatively low antioxidant capacity. Mitochondria and activated microglia are major sources of reactive oxygen species (ROS), which promote blood–brain barrier disruption, lipid peroxidation, and neuronal damage, including through peroxynitrite formation [50,52,53]. In parallel, activation of redox-sensitive pathways, including NF-κB and the NLRP3 inflammasome, further amplifies cytokine production and oxidative injury, reinforcing the bidirectional interaction between oxidative stress and inflammation.

Hemorrhagic stroke results from vessel rupture and bleeding into brain tissue, leading to primary mechanical damage and secondary injury driven by inflammation and oxidative stress [6,102]. Hemoglobin and heme released from erythrocytes induce ROS generation and ferroptosis and necroptosis, contributing to neuronal death. Microglia and infiltrating macrophages are rapidly activated. Although they initially facilitate hematoma clearance and tissue repair, persistent activation promotes excessive production of pro-inflammatory mediators and ROS, thereby aggravating secondary brain injury [95,112].

Collectively, available evidence supports a pivotal role of the redox–inflammation axis in both the initiation and progression of acute ischemic stroke, particularly during acute ischemia and reperfusion injury. Elevated concentrations of inflammatory cytokines and oxidative stress biomarkers have repeatedly been associated with larger infarct volume, greater neurological deficits, and poorer functional outcomes. However, the clinical utility of these biomarkers remains inconsistent across studies. While several investigations have reported significant associations between biomarkers such as IL-6, TNF-α, MPO, MDA, or F2-isoprostanes and stroke severity or prognosis, other studies have demonstrated weaker or non-significant relationships after adjustment for age, comorbidities, stroke subtype, timing of sample collection, and treatment strategies. These discrepancies likely reflect substantial heterogeneity in study design, patient populations, analytical methodologies, biological matrices, and sampling time points. Consequently, no single oxidative or inflammatory biomarker has yet demonstrated sufficient robustness for routine clinical decision-making in acute ischemic stroke. Future studies should prioritize standardized protocols, longitudinal biomarker assessment, and multimarker approaches integrating oxidative stress, inflammation, neuroimaging, and clinical variables to improve prognostic accuracy and facilitate personalized stroke management. 

#### Markers of Inflammation and Oxidative Stress in Stroke

Studies show that inflammatory processes and oxidative stress play a key role in the pathogenesis of acute ischemic stroke (AIS). The study by Ormstad et al. analyzed the serum levels of 13 cytokines and CRP in patients with AIS. Among 85 participants (45 with AIS, 40 healthy controls), significantly higher levels of IL-1ra (IL-1 receptor antagonist), IL-6, IL-18, and CRP were observed in patients with AIS compared with controls. Importantly, CRP levels correlated positively with the volume of cerebral infarction, suggesting its potential usefulness as an indicator of brain injury severity. IL-1β and TNF-α levels were not elevated, which the authors attribute to an early increase after stroke onset and a possible decline before sampling. Additionally, there has been a significant increase in the level of the chemokine GRO-α (CXCL1) in patients with AIS, particularly in those with radiologically confirmed infarctions, suggesting its potential usefulness as a marker of stroke etiology [15]. In another study, Chehaibi et al. analyzed the correlations among fasting glucose, antioxidant enzymes (glutathione peroxidase (GPx) and superoxide dismutase (SOD)), and inflammatory markers (hs-CRP and fibrinogen) in 196 patients with AIS. Patients were divided into groups with type 2 diabetes mellitus (T2DM) and without diabetes. A negative correlation was observed between higher fasting glucose levels and GPx and SOD levels in patients with T2DM. In contrast, positive correlations were observed between fasting glucose and hs-CRP and fibrinogen levels. These correlations persisted even after factors such as age, smoking, obesity, dyslipidemia, and hypertension were taken into account. Additionally, high CRP levels more than doubled the risk of stroke, regardless of the presence of diabetes. Reduced concentrations of GPx and SOD were associated with a higher risk of stroke, suggesting that hyperglycemia may lead to the progression of inflammation and increased oxidative stress, thereby increasing the risk of stroke [96].

The study by Tsai et al. evaluated the levels of oxidative stress markers, including malondialdehyde (MDA) and free thiols, in 100 patients with AIS compared with a control group. On days 1 and 7 after the onset of AIS, MDA levels were significantly elevated and free thiol levels were reduced compared with the control group, suggesting a role for oxidative stress in the pathogenesis of acute ischemic stroke. Patients were also divided into stroke subtypes: small–vessel stroke and large-vessel stroke. A significant difference was found in the concentrations of free thiols, which were significantly lower in the large vessel stroke group. MDA levels did not differ significantly between these groups, suggesting distinct pathogenetic mechanisms underlying the oxidation-antioxidant imbalance across stroke types. Additionally, MDA levels were independently associated with the patient’s clinical status at 3 months after stroke, with each 1 μM/L increase in MDA associated with a 37% increase in the risk of poor clinical status, suggesting a potential role for MDA as a predictive marker of AIS severity [113].

Additionally, new research has demonstrated the importance of other oxidative stress markers in AIS. For example, elevated levels of 4-hydroxynenal (4-HNE), a lipid peroxidation product, are associated with stroke recurrence, especially in patients with aspirin resistance. This suggests that 4-HNE may be a potential prognostic biomarker in acute ischemic stroke [114]. Reduced levels of glutathione (GSH)—a compound responsible for neutralizing free radicals can also be useful as an oxidative stress marker in AIS. The observed low GSH levels in acute ischemic stroke may be associated with poorer clinical prognosis and poorer functional outcomes in patients [101]. A very interesting study was also conducted by Milanligolu et al. They showed that patients with acute ischemic stroke showed significantly higher serum levels of malondialdehyde (MDA) within 24 h of stroke compared to the control group, indicating increased lipid peroxidation and increased oxidative stress. Catalase (CAT) activity, on the other hand, was significantly lower in stroke patients compared to healthy subjects. On the fifth day after the stroke, a further decrease in catalase and superoxide dismutase (SOD) activity was observed. On day 21, SOD activity remained subdued, while MDA, GSH-Px, and CAT levels showed no significant changes. However, there were no changes in GSH-Px and SOD activity during the first 24 h after stroke. This study suggests that patients with acute ischemic stroke experience increased oxidative stress and reduced antioxidant enzyme activity, which may play a role in the pathogenesis of stroke. Monitoring these parameters can help assess patients’ neurological status and predict their clinical outcomes [97].

Collectively, these findings indicate that acute ischemic stroke is characterized by a coordinated activation of inflammatory and oxidative pathways, rather than isolated biomarker changes. The observed alterations in cytokine levels, antioxidant enzyme activity, and lipid peroxidation products reflect a disruption of redox homeostasis, in which oxidative stress and inflammation mutually reinforce each other, contributing to neuronal injury and disease progression. Importantly, the dynamic nature of these biomarkers suggests their potential utility not only in diagnosis but also in monitoring disease severity and predicting clinical outcomes. Additional information on inflammatory and oxidative stress biomarkers in stroke is provided in Table 3.

## 8. Antioxidants

Antioxidants are compounds that protect cells from oxidative damage by neutralizing reactive oxygen species (ROS) and supporting endogenous redox defense systems [115]. They are broadly classified into endogenous (enzymatic and non-enzymatic) and exogenous antioxidants, the latter derived primarily from dietary sources [116].

### 8.1. Endogenous Antioxidants

The endogenous antioxidant system primarily relies on enzymatic defenses, including superoxide dismutase (SOD), catalase (CAT), glutathione peroxidase (GPx), and glutathione reductase (GR).

SOD serves as the first line of defense against oxidative stress by catalyzing the conversion of superoxide anion to hydrogen peroxide and oxygen [103,117]. Three isoforms—SOD1 (cytosolic), SOD2 (mitochondrial), and SOD3 (extracellular)—play complementary roles in maintaining redox homeostasis [102,118]. Importantly, SOD2 deficiency leads to severe mitochondrial dysfunction and cardiomyopathy, while SOD3 polymorphisms are associated with increased cardiovascular risk [119,120]. 

Catalase, localized mainly in peroxisomes, efficiently decomposes hydrogen peroxide into water and oxygen, preventing the formation of highly reactive hydroxyl radicals [104,121,122]. Experimental studies suggest that catalase exerts cardioprotective effects by limiting oxidative stress–induced myocardial remodeling [123].

The glutathione system, consisting of GPx and GR, plays a central role in intracellular redox regulation. GPx reduces hydrogen peroxide using reduced glutathione (GSH), while GR regenerates GSH from its oxidized form (GSSG), ensuring redox balance [100,105,110,111]. Impairment of this system has been associated with atherosclerosis and arrhythmias [124,125]. 

### 8.2. Exogenous Antioxidants

Exogenous antioxidants, mainly obtained from the diet, include polyphenols, flavonoids, and vitamins E and C [116,117].

Polyphenols, including resveratrol and quercetin, exert both direct antioxidant effects through radical scavenging and indirect effects via upregulation of endogenous antioxidant enzymes [126,127,128,129]. Resveratrol has been shown to reduce mitochondrial oxidative stress and improve endothelial function by activating eNOS [130,131], whereas quercetin may attenuate atherosclerotic plaque formation and improve vascular function [132].

Vitamin E acts as a lipid-soluble antioxidant that inhibits lipid peroxidation by neutralizing lipid radicals, thereby protecting cell membranes [133,134]. Vitamin C, a water-soluble antioxidant, directly scavenges ROS and regenerates vitamin E, enhancing overall antioxidant capacity [135]. Clinical and epidemiological studies suggest that higher vitamin C levels are associated with reduced cardiovascular risk and improved oxidative stress profiles [136,137].

Although exogenous antioxidants demonstrate protective effects in experimental models and selected clinical settings, their efficacy is influenced by bioavailability, dose, and interaction with endogenous systems, highlighting the complexity of antioxidant-based interventions in cardiovascular diseases. Additional information about endogenous and exogenous antioxidants, as well as major antioxidant and anti-inflammatory therapies, can be found in Table 4 and Table 5.

Beyond their direct antioxidant properties, several naturally occurring compounds, including resveratrol, quercetin, curcumin, sulforaphane, and epigallocatechin gallate (EGCG), have been shown to activate the Nrf2/Keap1 signaling pathway, thereby enhancing endogenous antioxidant defenses rather than acting solely as free radical scavengers. In parallel, several synthetic Nrf2 activators are currently under preclinical and early clinical investigation. These agents aim to restore redox homeostasis by activating endogenous cytoprotective pathways and may represent a promising direction for future precision-based cardiovascular therapies. Growing evidence suggests that pharmacological activation of the Nrf2/Keap1 pathway may attenuate oxidative stress, vascular inflammation, ferroptosis, and pyroptosis, making Nrf2 one of the most promising therapeutic targets currently under investigation in cardiovascular medicine [58,67,68,69].

### 8.3. Critical Perspective on Antioxidant Therapy in Cardiovascular Diseases

Despite compelling mechanistic evidence supporting the role of oxidative stress in cardiovascular pathophysiology, the clinical efficacy of antioxidant supplementation remains controversial. While experimental models consistently demonstrate protective effects of antioxidants against endothelial dysfunction, lipid peroxidation, and inflammatory activation, large randomized clinical trials in humans have produced inconsistent or neutral outcomes [138,139].

The discrepancy between strong experimental evidence and disappointing clinical outcomes likely results from several factors. First, many clinical trials tested broad antioxidant supplementation without confirming baseline oxidative stress or selecting patients with measurable redox imbalance. Second, most antioxidants used in large trials, including vitamins C and E and β-carotene, act as nonspecific radical scavengers and do not selectively target disease-relevant sources of ROS, such as mitochondria, NADPH oxidases, xanthine oxidase, or myeloperoxidase. Third, ROS are not exclusively harmful molecules; they also regulate vascular tone, mitochondrial adaptation, immune responses, and endogenous antioxidant defense. Therefore, indiscriminate ROS neutralization may interfere with physiological redox signaling. Fourth, differences in bioavailability, dose, treatment duration, disease stage, comorbidities, and concomitant pharmacotherapy may further explain inconsistent results across studies. Finally, most trials did not incorporate biomarker-guided patient stratification, making it difficult to identify subgroups that might benefit from targeted redox modulation [26,28,29].

One fundamental limitation lies in the oversimplified assumption that reactive oxygen species (ROS) are exclusively harmful. Under physiological conditions, ROS serve as essential signaling molecules that regulate vascular tone, angiogenesis, mitochondrial adaptation, and immune responses. Excessive and non-selective scavenging of ROS may therefore disrupt redox-sensitive signaling pathways, including those mediated by Nrf2, nitric oxide (NO), and mitochondrial biogenesis. This dual role of ROS suggests that oxidative stress in CVD is not merely a consequence of radical accumulation, but rather a dysregulation of redox homeostasis.

Another critical issue concerns bioavailability and pharmacokinetics. Many exogenous antioxidants, including polyphenols such as resveratrol or quercetin, demonstrate strong in vitro radical-scavenging capacity but undergo rapid metabolism and limited systemic bioavailability in vivo. Plasma concentrations achieved through dietary supplementation are often substantially lower than those used in experimental studies, raising questions about their true biological relevance.

Importantly, oxidative stress in cardiovascular diseases is frequently compartment-specific. Mitochondrial ROS, NADPH oxidase-derived superoxide, and myeloperoxidase-mediated oxidative pathways represent distinct sources of redox imbalance. Conventional antioxidant supplementation does not selectively target these localized sources, which may explain the limited clinical benefit observed with broad supplementation strategies.

Furthermore, patient heterogeneity must be considered. Baseline redox status, comorbidities such as diabetes or chronic kidney disease, genetic polymorphisms affecting antioxidant enzymes (e.g., SOD or GPx variants), and differences in inflammatory burden may all influence therapeutic response. Most large trials have not stratified patients by oxidative stress biomarkers, potentially obscuring subgroup-specific benefits.

Emerging evidence suggests that rather than direct radical scavenging, modulation of endogenous antioxidant defense systems may offer a more promising strategy. Activation of redox-sensitive transcription factors such as Nrf2, enhancement in glutathione metabolism, or inhibition of specific ROS-generating enzymes (e.g., NOX inhibitors) represent targeted approaches that preserve physiological redox signaling while reducing pathological oxidative overload.

Taken together, current evidence does not support non-specific antioxidant supplementation as a broadly effective strategy for cardiovascular prevention or therapy. Future approaches should instead focus on targeted redox modulation, selection of patients with documented redox imbalance, and validation in adequately powered biomarker-guided clinical trials.

### 8.4. Redox Hormesis and Exercise-Induced ROS: Rethinking Antioxidant Strategies

An important concept increasingly recognized in cardiovascular redox biology is hormesis, defined as a biphasic response to stressors in which low-to-moderate levels of reactive oxygen species (ROS) exert adaptive and protective effects. In contrast, excessive levels become deleterious [145,146,147]. In this context, ROS should not be viewed solely as harmful byproducts of metabolism but rather as critical signaling molecules that regulate cellular adaptation, mitochondrial biogenesis, and the activation of antioxidant defenses.

Physical exercise represents one of the most compelling examples of redox hormesis. Acute exercise transiently increases ROS production, primarily from mitochondrial respiration and NADPH oxidases. However, this controlled oxidative stimulus activates adaptive pathways, including Nrf2-mediated transcription of antioxidant enzymes such as superoxide dismutase (SOD), catalase (CAT), and glutathione peroxidase (GPx) [147,148]. Repeated exposure to moderate elevations in ROS during regular exercise enhances endogenous antioxidant capacity, improves endothelial function, and reduces long-term cardiovascular risk.

Importantly, several studies suggest that high-dose antioxidant supplementation (e.g., vitamins C and E) may blunt exercise-induced beneficial adaptations by attenuating ROS-mediated signaling required for mitochondrial and metabolic remodeling [147,149]. These findings further support the notion that complete ROS suppression is neither physiologically desirable nor therapeutically optimal. Instead, cardiovascular prevention strategies should aim to restore redox balance rather than eliminate ROS.

### 8.5. Genetic Variability in Antioxidant Systems: The Example of SOD3

Genetic polymorphisms affecting endogenous antioxidant enzymes may partially explain interindividual variability in oxidative stress burden and cardiovascular risk. Among these, variants in the SOD3 gene, encoding extracellular superoxide dismutase (ecSOD), have attracted particular attention.

EcSOD plays a crucial role in protecting the vascular extracellular matrix from superoxide-mediated inactivation of nitric oxide (NO), thereby preserving endothelial function. The R213G polymorphism in SOD3 alters the enzyme’s heparin-binding domain, leading to redistribution of ecSOD from the vascular wall to the circulation and reduced local antioxidant protection [119].

The Copenhagen City Heart Study demonstrated that carriers of the R213G variant exhibited an increased risk of ischemic heart disease compared to non-carriers, suggesting a clinically relevant impact of impaired extracellular antioxidant defense [119]. These findings highlight the importance of considering genetic background when evaluating oxidative stress and therapeutic responsiveness.

Incorporating genetic profiling of antioxidant enzyme polymorphisms, including SOD3, GPx, and catalase, may, in the future, refine cardiovascular risk stratification and support the development of personalized redox-modulating interventions.

## 9. Antioxidants and Anti-Inflammatory Therapy in the Treatment and Prevention of Cardiovascular Diseases

### 9.1. Experimental Evidence

The antioxidant properties of many plant-derived compounds, as well as those of endogenous enzymes in the human body, suggest their potential use as preventive or supportive therapies for cardiovascular diseases (CVDs).

The meta-analysis by Wan et al. [139] included 284 randomized clinical trials involving 17,613 participants. It aimed to assess the effects of polyphenol supplementation on CVD risk factors, including blood pressure, lipid profile, and glycemic parameters. The analysis showed that supplementation with genistein, resveratrol, and catechins contributed to lowering blood pressure, with the greatest effect recorded for genistein—a reduction of 10 mmHg for systolic blood pressure and 9 mmHg for diastolic pressure. In terms of lipid profile, anthocyanins were the most effective, lowering LDL, triglyceride (TG), and total cholesterol (TC) levels, while increasing HDL concentration. Flavonoids and genistein also showed beneficial effects on lipid metabolism. In terms of glycemic parameters, curcumin improved all three indicators analyzed: fasting blood glucose, insulin levels, and glycated hemoglobin (HbA1c). Flavonols and genistein, on the other hand, had a positive effect on glycemia and insulin levels but did not significantly affect HbA1c [139].

The results of this meta-analysis indicate significant potential for polyphenols in reducing CVD risk factors; however, the authors emphasized the need for further research to determine the efficacy and optimal duration of supplementation more accurately [14].

A meta-analysis by An et al., which included 884 randomized trials involving more than 883,000 participants, comes to similar conclusions. It evaluated the effect of micronutrient supplementation with antioxidant properties on the incidence of heart attack, stroke, and type 2 diabetes, as well as on selected metabolic parameters, including blood pressure, lipid profile, and glucose levels. The analysis showed that omega-3 fatty acids significantly reduce cardiovascular mortality and reduce the incidence of heart attack and coronary artery disease. Folic acid supplementation was associated with a reduced risk of stroke, and coenzyme Q10 with a reduced risk of overall mortality. According to previous findings, genistein had the strongest effect in lowering blood pressure, and curcumin showed beneficial effects on HbA1c levels, fasting glycemia, and insulin levels. Importantly, there was no positive effect of vitamin C or E supplementation on the incidence of CVD, while β-carotene supplementation was associated with an increased risk of stroke and an increase in overall and cardiovascular mortality [112].

These results highlight that not all forms of antioxidant supplementation provide clinical benefit. In some cases, such as β-carotene, it can even lead to negative health effects, underscoring the need for individualized supplementation and a cautious, evidence-based approach.

Further relevant data are provided by the COSMOS study, conducted by Sesso et al., which involved more than 21,000 people aged 60 (men) and 65 (women). The subjects were given supplements containing cocoa flavonoids. After 3.6 years of follow-up, it was associated with a 27% reduction in this group. In addition, regular supplementation—with at least 75% of doses taken– has contributed to a 15% reduction in the overall number of cardiovascular events, confirming the importance of systematic supplementation for achieving therapeutic benefits [150]. Although these findings are encouraging, they should be interpreted with caution, as the observed benefits may reflect pleiotropic biological effects of cocoa flavonoids rather than direct antioxidant activity alone. Further randomized studies are required to confirm these observations.

Recent evidence further suggests that pharmacological agents not primarily developed as antioxidants may indirectly attenuate oxidative stress and chronic inflammation by improving metabolic homeostasis. Also worth mentioning is the SELECT study conducted by Deanfield et al. [151], which included more than 17,000 overweight patients with diagnosed cardiovascular disease. The study aimed to determine the effect of semaglutide (Ozempic)—a drug previously used mainly to treat obesity and diabetes—on the risk of serious cardiovascular events. The medication was administered once a week for about 3.5 years. The results showed that the use of semaglutide in this population significantly reduced the risk of CVD-related deaths and complications, suggesting its potential new use in the primary and secondary prevention of cardiovascular disease [151]. Beyond lipid-lowering, statins exert well-documented pleiotropic effects, including modulation of oxidative stress and inflammation. Experimental and clinical evidence indicates that statins improve endothelial function, reduce NADPH oxidase activity, enhance nitric oxide bioavailability, suppress NF-κB-mediated inflammatory signaling, and may activate Nrf2-dependent antioxidant responses. These mechanisms contribute to plaque stabilization and reduction in cardiovascular events independently of LDL cholesterol lowering. Consequently, statins are among the best-established examples of clinically effective pharmacological modulation of the redox–inflammation axis [152,153,154,155]. Table 6 presents additional information on major randomized clinical trials evaluating antioxidant, redox-modulating, and anti-inflammatory therapies in cardiovascular diseases.

Collectively, these studies demonstrate that cardiovascular benefit depends more on selective modulation of disease-relevant inflammatory and redox pathways than on non-specific antioxidant supplementation, supporting a paradigm shift toward mechanism-based therapeutic strategies.

Overall, the available evidence indicates that the disappointing results of conventional antioxidant supplementation do not invalidate the role of oxidative stress in cardiovascular diseases. Rather, they highlight the complexity of redox biology and suggest that broad radical scavenging is insufficient to modify disease progression. Emerging evidence increasingly supports targeted modulation of redox-sensitive and inflammatory pathways, combined with biomarker-guided patient stratification, as a more promising therapeutic strategy for precision cardiovascular medicine.

### 9.2. Why Have Antioxidant Therapies Failed?

Although numerous experimental studies have demonstrated beneficial effects of antioxidant compounds, the translation of these findings into clinical practice has been largely disappointing. Large randomized clinical trials evaluating non-specific antioxidant supplementation, particularly vitamins C and E and β-carotene, have generally failed to demonstrate consistent reductions in cardiovascular morbidity or mortality and, in some cases, have even suggested potential harm [138].

Several explanations have been proposed for these discrepancies. First, most antioxidants evaluated in clinical trials act as non-specific radical scavengers and do not selectively target the principal enzymatic sources of pathological ROS, such as NADPH oxidases, xanthine oxidase, or dysfunctional mitochondria. Second, ROS also serve essential physiological signaling functions in vascular homeostasis, immune regulation, and cellular adaptation; therefore, indiscriminate ROS scavenging may disrupt normal redox signaling. Third, oxidative stress is highly dynamic and heterogeneous, varying according to disease stage, tissue compartment, comorbidities, and individual patient characteristics. Finally, most clinical trials did not incorporate biomarker-guided patient selection or redox phenotyping, making it unlikely that patients with the highest oxidative burden were specifically targeted. Consequently, beneficial effects observed in selected patient subgroups may have been diluted in heterogeneous study populations, potentially contributing to the inconsistent clinical outcomes. 

Despite encouraging mechanistic and preclinical evidence, many redox-modulating strategies have not yet demonstrated consistent clinical benefit in large randomized trials. Therefore, future therapeutic approaches should be interpreted cautiously until supported by robust prospective evidence and validated biomarker-guided patient selection.

Future therapeutic strategies are likely to benefit from targeted modulation of redox-sensitive signaling pathways; however, their clinical efficacy remains to be confirmed in adequately powered prospective trials.

Other targeted redox-modulating approaches, including NADPH oxidase inhibitors, Nrf2 activators, xanthine oxidase inhibitors, and mitochondria-targeted antioxidants, have demonstrated encouraging preclinical results; however, robust evidence from large randomized cardiovascular outcome trials remains limited [11,49].

## 10. Future Perspectives and Research Directions

Despite significant advances in understanding the role of oxidative stress and inflammation in cardiovascular diseases (CVDs), important translational gaps remain. Although numerous inflammatory and oxidative biomarkers have been identified, their integration into routine clinical decision-making is still limited. Most studies rely on single markers, such as hs-CRP or malondialdehyde (MDA), which insufficiently capture the complexity of the redox–immune network driving vascular pathology. Future research should therefore prioritize multimarker strategies integrating inflammatory cytokines, lipid peroxidation products, antioxidant enzyme activity, and composite indices such as the oxidative stress index (OSI). Longitudinal profiling rather than a single time-point assessment may significantly improve risk stratification and prognostic accuracy.

Another major challenge concerns the dual role of reactive oxygen species (ROS). While excessive ROS contribute to endothelial dysfunction, LDL oxidation, and plaque destabilization, physiological redox signaling is essential for vascular homeostasis. The lack of consistent benefit observed in large randomized trials of antioxidant supplementation suggests that indiscriminate ROS scavenging may disrupt adaptive redox pathways. Future mechanistic investigations should differentiate pathological oxidative overload from physiological signaling, focus on compartment-specific ROS sources (e.g., mitochondrial versus NADPH oxidase-derived), and explore the therapeutic modulation of redox-sensitive transcription factors such as Nrf2 and NF-κB.

Emerging evidence also highlights the importance of immunometabolism and novel molecular biomarkers in cardiovascular pathology. Beyond conventional inflammatory cytokines and oxidative stress markers, components of the NLRP3 inflammasome (including NLRP3, ASC, caspase-1, IL-18, and IL-1β), extracellular vesicles (particularly endothelial- and platelet-derived exosomes), and circulating microRNAs (e.g., miR-21, miR-126, miR-146a, and miR-155) have emerged as promising diagnostic and prognostic biomarkers reflecting vascular inflammation, endothelial dysfunction, and plaque instability. Beyond their diagnostic potential, several of these molecules—including the NLRP3 inflammasome, specific microRNAs, and extracellular vesicle-mediated signaling pathways—also represent promising therapeutic targets that warrant further experimental and clinical investigation. In parallel, metabolomic and lipidomic signatures are increasingly recognized as complementary tools capable of capturing systemic metabolic disturbances associated with cardiovascular disease. Integration of these emerging biomarkers with redox biology, mitochondrial function, imaging modalities, and artificial intelligence- and machine-learning-based analytical platforms has the potential to improve cardiovascular risk prediction, provided appropriate analytical validation and prospective clinical evaluation are conducted. Although multimarker strategies, system biology, and artificial intelligence represent promising directions for cardiovascular risk stratification, prospective clinical validation remains limited. Most proposed biomarker panels and AI-based prediction models require external validation, analytical standardization, and demonstration of incremental predictive value beyond established cardiovascular risk factors before routine clinical implementation can be recommended. Furthermore, heterogeneity in biological matrices, analytical platforms, and pre-analytical procedures currently limits direct comparisons between studies and remains a major barrier to the clinical implementation of multimarker approaches. Importantly, these complementary technologies may capture biological processes that cannot be adequately reflected by conventional inflammatory or oxidative stress markers alone. Inflammasome-related biomarkers reflect activation of innate immunity; extracellular vesicles enable assessment of intercellular communication and endothelial injury; and circulating microRNAs and metabolomic signatures provide insight into post-transcriptional regulation and systemic metabolic remodeling. Together, these complementary approaches may substantially improve disease phenotyping and facilitate personalized cardiovascular risk prediction. Nevertheless, widespread clinical adoption will ultimately depend on robust multicenter validation, harmonized analytical protocols, and demonstration of clinically meaningful incremental value beyond established cardiovascular risk assessment tools.

Precision-based therapeutic strategies represent another key direction. Heterogeneous responses to antioxidant supplementation indicate that therapeutic efficacy likely depends on baseline redox status, comorbidities, and genetic background. Biomarker-guided patient stratification based on oxidative and inflammatory profiles may improve the effectiveness of targeted interventions. Furthermore, therapies with combined anti-inflammatory and redox-modulating properties—such as IL-1β pathway inhibition or cardiometabolic agents with pleiotropic effects—deserve further investigation in well-designed prospective trials.

Ultimately, successful clinical translation will require large, multicenter prospective studies to validate multimarker panels, harmonize analytical methodologies, and determine whether targeted modulation of the redox–inflammation axis translates into meaningful reductions in cardiovascular morbidity and mortality. Integrating mechanistic insights with validated biomarkers, multi-omics technologies, and artificial intelligence-based analytical platforms may provide the foundation for future precision cardiovascular medicine.

### Limitations of This Review

Several limitations of the present review should be acknowledged. First, the available evidence is characterized by substantial heterogeneity in study designs, patient populations, cardiovascular disease subtypes, biological matrices, analytical methods, and the timing of biomarker assessment, which limits direct comparisons across studies. Second, many oxidative stress and inflammatory biomarkers lack standardized analytical protocols, universally accepted cut-off values, and inter-laboratory harmonization, thereby restricting their routine clinical implementation. Third, although numerous biomarkers have demonstrated promising diagnostic and prognostic associations, relatively few have undergone prospective validation or demonstrated incremental value beyond established cardiovascular risk factors. Finally, the translational interpretation of redox biology remains challenging because reactive oxygen species exert both physiological and pathological functions, while most clinical intervention studies have evaluated non-specific antioxidant supplementation rather than mechanism-based modulation of disease-relevant redox pathways. Consequently, the conclusions presented in this review should be interpreted in light of these methodological and translational limitations, which further underscore the need for standardized biomarker validation, biomarker-guided patient stratification, and well-designed prospective clinical trials.

## 11. Conclusions

Cardiovascular diseases are increasingly recognized as disorders driven by a complex interplay between oxidative stress and chronic inflammation, in addition to classical cardiovascular risk factors. The redox–inflammation axis is a dynamic, self-amplifying network linking endothelial dysfunction, immune activation, lipid oxidation, plaque instability, and ischemic injury, thereby contributing to the initiation and progression of atherosclerosis and stroke.

Although substantial progress has been made in understanding the molecular mechanisms underlying these processes, their clinical translation remains incomplete. Current oxidative stress and inflammatory biomarkers provide valuable mechanistic and prognostic information but are limited by insufficient specificity, biological variability, and lack of analytical standardization. Similarly, the inconsistent results of conventional antioxidant supplementation indicate that non-specific ROS scavenging is insufficient to modify cardiovascular outcomes and underscore the complexity of redox biology.

This review highlights a conceptual shift from viewing oxidative stress as excessive free radical production toward understanding it as dysregulation of finely controlled redox signaling. This perspective provides a more comprehensive framework for interpreting previous clinical failures and supports the development of mechanism-based therapeutic strategies targeting disease-relevant redox and inflammatory pathways.

Future advances in cardiovascular medicine will likely depend on integrating multimarker profiling, redox biology, immunometabolism, and system-level approaches with precision medicine strategies. Improved patient stratification, validation of emerging biomarkers, and targeted modulation of the redox–inflammation axis may ultimately enable more effective prevention, risk prediction, and personalized treatment of cardiovascular diseases.

In conclusion, this review provides an integrated perspective on the redox–inflammation axis by linking mechanistic insights with biomarker evaluation and translational challenges in atherosclerosis and acute ischemic stroke. This framework may facilitate the development of biomarker-guided precision medicine strategies, improve cardiovascular risk stratification, and support the design of targeted redox-modulating therapies. Future progress will depend on robust biomarker standardization, prospective clinical validation, and interdisciplinary approaches integrating molecular biology, system medicine, multi-omics, and artificial intelligence.

## Figures and Tables

**Table 1:** Comparative diagnostic and prognostic utility of major oxidative stress and inflammatory biomarkers in cardiovascular diseases.

Biomarker	Biological Significance	Diagnostic Utility	Prognostic Utility	Current Clinical Applicability	Major Limitations	Key References
hs-CRP	Systemic inflammation	Well established	Well established	Routine clinical use	Low disease specificity; reflects systemic rather than vascular inflammation	[3,88,91]
IL-6	Pro-inflammatory cytokine	Promising	Strong evidence	Investigational	Biological variability; lack of standardized cut-off values	[62,64,65,92]
Myeloperoxidase (MPO)	Oxidative inflammation and neutrophil activation	Emerging	Promising	Research	Limited assay standardization; influenced by systemic inflammatory conditions	[95]
Malondialdehyde (MDA)	Lipid peroxidation	Emerging	Moderate evidence	Research	Low specificity; susceptible to analytical variability	[87,96,97]
F2-Isoprostanes	Oxidative lipid damage	Promising	Moderate evidence	Research	Expensive analytical methods; limited routine availability	[94]
Oxidized LDL (oxLDL)	Lipoprotein oxidation and atherosclerosis	Emerging	Moderate evidence	Research	Lack of assay harmonization; influenced by metabolic status	[86,98,99]
Reduced/Oxidized glutathione (GSH/GSSG)	Cellular redox balance	Emerging	Emerging	Experimental	Pre-analytical instability; methodological variability	[74,100,101]
Antioxidant enzymes (SOD, CAT, GPx)	Endogenous antioxidant defense	Promising	Moderate evidence	Research	High biological variability; lack of standardized reference ranges	[102,103,104,105]

Abbreviations: hs-CRP, high-sensitivity C-reactive protein; IL-6, interleukin-6; MPO, myeloperoxidase; MDA, malondialdehyde; oxLDL, oxidized low-density lipoprotein; GSH/GSSG, reduced/oxidized glutathione; SOD, superoxide dismutase; CAT, catalase; GPx, glutathione peroxidase.

**Table 2:** Key inflammatory and oxidative stress biomarkers in atherosclerosis.

Biomarker	Type	Source/Mechanism	Clinical/Pathophysiological Role	Limitations	References
hs-CRP	Inflammatory	Synthesized in liver in response to IL-6	Marker of systemic inflammation; predictor of CVD events	Low specificity; reflects inflammation rather than causation	[87,91]
IL-6	Inflammatory	Produced by immune and vascular cells; activates JAK/STAT pathway	Promotes CRP synthesis; associated with plaque progression and future CV events	Influenced by systemic conditions, not disease-specific	[92]
suPAR	Inflammatory	Released from immune cells (urokinase receptor cleavage)	Independent predictor of cardiovascular events	Limited standardization in clinical use	[89]
SAA	Inflammatory	Acute-phase protein produced in liver	Involved in plaque destabilization	Low specificity; elevated in many inflammatory states	[90]
MPO	Inflammatory/Oxidative	Released by neutrophils; generates ROS	Promotes LDL oxidation and plaque instability	Reflects neutrophil activation rather than specific vascular damage	[95]
IL-1β	Inflammatory	Produced via inflammasome activation	Early mediator of inflammation; contributes to endothelial dysfunction	Highly dynamic; difficult to standardize	[93]
MDA	Oxidative	Product of lipid peroxidation	Correlates with disease severity and myocardial injury	Low specificity; influenced by diet and metabolism	[87]
F2-isoprostanes	Oxidative	Non-enzymatic oxidation of arachidonic acid	Reliable marker of in vivo oxidative stress	Requires advanced analytical methods	[94]
13-HODE	Oxidative	Oxidation product of linoleic acid	Involved in lipid metabolism and foam cell formation	Less standardized; context-dependent interpretation	[54,57]
oxLDL	Oxidative/Lipid	Oxidized LDL particles	Key driver of foam cell formation and plaque progression	Measurement variability; assay differences	[54,57]
NO (↓ bioavailability)	Endothelial/Redox	Produced by eNOS; reduced under oxidative stress	Marker of endothelial dysfunction	Indirect measurement; influenced by multiple pathways	[13,47,48]
GSH/GSSG ratio	Redox	Intracellular glutathione system	Indicator of cellular redox balance	Requires specialized assays; intracellular variability	[48,100,110,111]

The downward arrow (↓) indicates decreased bioavailability.

**Table 3:** Key inflammatory and oxidative stress biomarkers in acute ischemic stroke (AIS).

Biomarker	Type	Source/Mechanism	Clinical/Pathophysiological Role	Limitations	References
CRP (hs-CRP)	Inflammatory	Synthesized in the liver in response to IL-6	Correlates with infarct volume; predictor of stroke severity and risk	Low specificity; reflects systemic inflammation	[15,96]
IL-6	Inflammatory	Produced by immune and vascular cells	Promotes inflammatory response; associated with stroke progression	Influenced by comorbidities (e.g., diabetes)	[15]
IL-18	Inflammatory	Produced via inflammasome activation	Involved in immune activation and neuroinflammation	Dynamic expression; time-dependent variability	[15]
IL-1ra	Inflammatory (regulatory)	Natural antagonist of IL-1 receptor	Reflects activation of the IL-1 signaling pathway	Interpretation complex (protective vs. compensatory)	[15]
GRO-α (CXCL1)	Chemokine	Produced by activated immune cells	Associated with stroke etiology and inflammatory recruitment	Limited clinical validation	[15]
Fibrinogen	Inflammatory/Coagulation	Acute-phase protein	Marker of systemic inflammation; linked to thrombosis	Non-specific; influenced by multiple factors	[96]
MDA	Oxidative	Product of lipid peroxidation	Correlates with stroke severity and poor clinical outcomes	Low specificity; influenced by metabolic status	[97,113]
Free thiols	Oxidative	Reflect systemic redox status (antioxidant capacity)	Decreased levels associated with severe stroke subtypes	Sensitive to systemic conditions	[113]
4-HNE	Oxidative	Lipid peroxidation product	Associated with stroke recurrence and aspirin resistance	Limited standardization	[114]
GSH (↓ levels)	Redox	Intracellular antioxidant	Low levels linked to worse prognosis and oxidative imbalance	Intracellular measurement variability	[101]
GPx	Antioxidant enzyme	Glutathione-dependent enzyme	Reduced activity associated with increased stroke risk	Influenced by metabolic status (e.g., hyperglycemia)	[96,97]
SOD	Antioxidant enzyme	Converts superoxide radicals	Reduced activity reflects impaired antioxidant defense	Time-dependent variability post-stroke	[96,97]
CAT	Antioxidant enzyme	Decomposes hydrogen peroxide	Decreased activity indicates oxidative imbalance	Dynamic changes over time	[97]

Abbreviations: CRP, C-reactive protein; hs-CRP, high-sensitivity C-reactive protein; IL, interleukin; GRO-α (CXCL1), growth-regulated oncogene alpha; MDA, malondialdehyde; 4-HNE, 4-hydroxynonenal; GSH, glutathione; GPx, glutathione peroxidase; SOD, superoxide dismutase; CAT, catalase. The downward arrow (↓) indicates decreased levels.

**Table 4:** Endogenous and exogenous antioxidants.

Name	Origin	Mechanism of Action
Superoxide dismutase (SOD)	Endogenous	Conversion of superoxide anion radical into hydrogen peroxide and oxygen 2O_2_^−^ + 2H^+^ 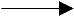 H_2_O_2_ + O_2_ [103]
Catalase (CAT)	Endogenous	Attachment of hydrogen peroxide and formation of compound I (Cpd I—compound one), and reaction with the second molecule H_2_O_2,_ and release of the product Enz (Por-Fe^III^) + H_2_O_2_ 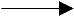 Cpd I (Por·^+^-Fe^IV^=O) + H_2_O cpd i (cf^+^-Fe^IV^=O) + H_2_O_2_ 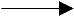 Enz (Por-Fe^III)^ + H_2_O + O_2_ [121]
Glutathione peroxidase	Endogenous	RSeH + H_2_O_2_ 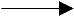 RSeOH + H_2_O RSeOH + GSH 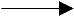 GS-SeR + H_2_O GS-SeR + GSH 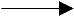 GSSG + RSeH GSSG + NADPH + H^+^ 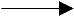 2 GSH + NADP^+^ (GRx Reaction) [111]
Glutathione reductase	Endogenous	RSeH + H_2_O_2_ 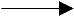 RSeOH + H_2_O RSeOH + GSH 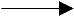 GS-SeR + H_2_O GS-SeR + GSH 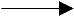 GSSG + RSeH GSSG + NADPH + H^+^ 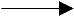 2 GSH + NADP^+^ (GRx Reaction) [111]
Resveratrol	Exogenous	Directly as an antioxidant—using hydroxyl groups, or indirectly—inducing the expression of many antioxidant enzymes [128,129]
Quercetin	Exogenous	Use of hydroxyl groups (similar to resveratrol) [126,127]
Vitamin E	Exogenous	Reaction with lipid radicals—formed during lipid peroxidation, transfer of a hydrogen atom, and transformation of the radical to its less reactive form [133]
Vitamin C	Exogenous	Direct—inhibition of the formation of radicals such as the hydroxyl radical—possible owing to the ability to transfer electrons, which allows for neutralization of RFT and RFA Indirectly—increasing α-tocopherol, thus enhancing the antioxidant effect of vitamin E [135]

**Table 5:** Major antioxidant and anti-inflammatory therapies targeting the redox–inflammation axis in cardiovascular diseases: mechanisms of action and current clinical evidence.

Antioxidant/Therapy	Main Anti-Inflammatory/Redox Mechanism	Principal CVD	Clinical Evidence	Key References
Vitamin C	ROS scavenging	Atherosclerosis	Inconsistent benefit in RCTs	[138,139]
Vitamin E	Inhibition of lipid peroxidation	Atherosclerosis	Negative or neutral RCTs (HOPE, HOPE-TOO)	[55,140]
Polyphenols (resveratrol, curcumin, flavonoids)	Nrf2 activation, NF-κB inhibition	Atherosclerosis	Promising effects on surrogate biomarkers	[25,26,58,67,68]
Omega-3 fatty acids	Cytokine modulation, resolution of inflammation	CAD	Moderate evidence for cardiovascular benefit	[56]
Statins	NF-κB inhibition, Nrf2 activation, improved endothelial function	CAD/Stroke	Strong clinical evidence	[81,99,117,141]
Colchicine	NLRP3 inflammasome inhibition	CAD	Strong evidence (COLCOT, LoDoCo2)	[142,143]
Canakinumab	IL-1β inhibition	CAD	Proven reduction in recurrent cardiovascular events (CANTOS)	[144]

**Table 6:** Major randomized clinical trials evaluating antioxidant, redox-modulating, and anti-inflammatory therapies in cardiovascular diseases.

Trial/Intervention	Target	Disease Population	Main Findings	Clinical Implication
HOPE (Vitamin E) [140]	Non-specific antioxidant	High cardiovascular risk	No reduction in major cardiovascular events	Does not support routine antioxidant supplementation
HOPE-TOO (Vitamin E) [55]	Non-specific antioxidant	High cardiovascular risk	No long-term cardiovascular benefit	Negative
CHAOS (Vitamin E) [156]	Antioxidant	Coronary artery disease	Reduced non-fatal myocardial infarction, but no mortality benefit	Limited clinical value
COSMOS (Cocoa flavanols) [150]	Polyphenols/indirect redox modulation	Older adults	Reduced cardiovascular mortality and events in adherent participants	Promising; requires confirmation
Meta-analysis (Wan et al.) [139]	Multiple antioxidant mechanisms (polyphenols)	Cardiovascular risk factors	Improved blood pressure, lipid profile, and glycemic control	Favorable effects on surrogate cardiovascular risk factors
Meta-analysis (An et al.) [138]	Micronutrients	General population	Omega-3 fatty acids and folic acid beneficial; vitamins C and E ineffective; β-carotene associated with adverse outcomes	Benefits depend on intervention rather than antioxidant activity alone
Allopurinol [155]	Xanthine oxidase inhibition	CVD/endothelial dysfunction	Improved endothelial function and reduced oxidative stress; inconsistent effects on major cardiovascular outcomes	Mechanistically promising; insufficient evidence for routine cardiovascular use
Nrf2 activators [22,154]	Nrf2/Keap1 pathway	Experimental/preclinical	Enhanced endogenous antioxidant defenses and reduced oxidative stress	Promising therapeutic target; clinical evidence remains limited
Mitochondria-targeted antioxidants (e.g., MitoQ) [22]	Mitochondrial ROS	Early clinical studies	Improved oxidative stress markers and endothelial function; limited cardiovascular outcome data	Requires larger randomized clinical trials
SELECT (Semaglutide) [151]	Metabolic and inflammatory modulation	Overweight patients with established CVD	Reduced major adverse cardiovascular events	Cardiovascular benefit is likely mediated by pleiotropic metabolic and anti-inflammatory effects rather than direct antioxidant activity
CANTOS (Canakinumab) [144]	IL-1β inhibition	Previous myocardial infarction with residual inflammation	Reduced recurrent cardiovascular events	Proof-of-concept for targeted anti-inflammatory therapy
COLCOT (Colchicine) [143]	Inflammation	Recent myocardial infarction	Reduced recurrent ischemic events	Supports inflammation-targeted therapy
LoDoCo2 (Colchicine) [142]	Inflammation	Chronic coronary artery disease	Reduced cardiovascular events	Confirms the benefit of targeted anti-inflammatory treatment

Abbreviations: CVD, cardiovascular disease; ROS, reactive oxygen species; Nrf2, nuclear factor erythroid 2-related factor 2; IL-1β, interleukin-1β.

## Data Availability

No new data were created or analyzed in this study. Data sharing is not applicable to this article.
